# Trends and cross-country inequalities in the global burden of neurodevelopmental disorders among children aged 0–14 from 1990 to 2021

**DOI:** 10.3389/fpubh.2025.1609254

**Published:** 2025-09-01

**Authors:** Tian Jia, Yezi Kong, Guna Zhao, Yu Wang

**Affiliations:** ^1^Department of Child Healthcare, Northwest University First Hospital, Xi’an, Shaanxi, China; ^2^Clinical Medical School, Haiyuan College, Kunming Medical University, Kunming, Yunnan, China; ^3^Department of Pediatrics, Northwest University First Hospital, Xi’an, Shaanxi, China; ^4^Department of Pharmacy, Northwest University First Hospital, Xi’an, Shaanxi, China

**Keywords:** neurodevelopmental disorders, children, health inequalities, prevalence, trends, prediction

## Abstract

**Background:**

To evaluate the trends and cross-country inequalities of three common neurodevelopmental disorders (NDDs): Autism spectrum disorders (ASD), Attention-deficit/hyperactivity disorder (ADHD) and Idiopathic developmental intellectual disability (IDID) among children aged 0–14, and further predicted its changes to 2046.

**Methods:**

The estimates and their 95% uncertainty interval (UI) for prevalence of ASD, ADHD and IDID among children aged 0–14 across 204 countries were extracted from Global Burden of Disease (GBD) 2021. Joinpoint regression analysis was used to calculate the average annual percentage changes (AAPC). The slope index of inequality (SII) and concentration index recommended by the World Health Organization (WHO) are two standard indicators for measuring absolute and relative gradient inequality. Our study used these two indicators to quantify the inequality of this three common NDDs burden between countries with different Sociodemographic Index (SDI). Finally, we used the Nordpred model to predict the disease burden of NDDs in 2046.

**Results:**

The AAPC (95% confidence interval [CI]) in prevalence of the three common NDDs among children aged 0–14 worldwide from 1990 to 2021 were as follows: ASD 0.09 (0.08 to 0.09), ADHD −0.08 (−0.12 to −0.04) and IDID −0.86 (−0.88 to −0.84). The SII (95% CI) changed from 27.09 (−29.98 to 84.17) in 1990 to 38.36 (−21.48 to 98.20) in 2021 for ASD, from 1402.78 (1100.25 to 1705.31) in 1990 to 1402.76 (1083.55 to 1721.97) in 2021 for ADHD, from −594.52 (−755.05 to −434.00) in 1990 to −545.94 (−673.19 to −418.69) in 2021 for IDID. The concentration index (95% CI) showed 0.15 (0.07 to 0.23) in 1990 and 0.19 (0.10 to 0.26) in 2021 for ASD, 0.07 (−0.02 to 0.16) in 1990 and 0.02 (−0.07 to 0.11) in 2021 for ADHD, 0.44 (0.34 to 0.53) in 1990 and 0.39 (0.28 to 0.48) in 2021 for IDID. Compared to 2021, the age-standardized prevalence rates (ASPR) in 2046 of the three common NDDs showed a slight decrease in ASD and ADHD, a slight increase in IDID.

**Conclusion:**

As a major public health concern, the global burden of NDDs in children exhibited distinct trends from 1990 to 2021: an increasing trend for ASD, and decreasing trends for ADHD and IDID. Health inequalities persist across these conditions. The burdens of ASD and ADHD are primarily concentrated in high-SDI countries/territories, whereas the burden of IDID is more prevalent in low-SDI countries/territories. Therefore, targeted public health strategies and equitable allocation of healthcare resources are essential to effectively mitigate the burden of NDDs.

## 1 Introduction

Neurodevelopmental disorders (NDDs), a group of clinically heterogeneous conditions characterized by impairments in brain development, cognitive function, and behavioral regulation, impose lifelong challenges on affected individuals and families (1). The fifth edition of the Diagnostic and Statistical Manual of Mental Disorders (DSM-5) classify autism spectrum disorder (ASD), Attention-deficit/hyperactivity disorder (ADHD), Idiopathic developmental intellectual disability (IDID), specific learning disabilities, and movement disorders (such as developmental coordination disorder and tic disorders) as NDDs (2). ASD is a neurodevelopmental disorder characterized by persistent deficits in social communication and social interaction across multiple contexts, accompanied by restricted, repetitive patterns of behavior, interests, or activities (3). ADHD is defined by a persistent pattern of inattention and/or hyperactivity-impulsivity that interferes with functioning or development (4). IDID refers to intellectual disability of unknown etiology, characterized by significant limitations in both intellectual functioning and adaptive behavior that manifest during the developmental period (5, 6). In 2021, the global number of cases for three common NDDs—ASD, ADHD, and IDID—was estimated at approximately 235 million (7). The associated burdens extend far beyond direct healthcare costs, encompassing substantial expenditures on special education, productivity losses among caregivers, and profound psychosocial impacts, including stigma, familial distress, and reduced quality of life (8). There is also evidence that early-life experiences can have profound effects on brain development and behavior, and their impact on quality of life across the lifespan should not be overlooked (9). Therefore, it is crucial to understand the characteristics of the disease burden associated with NDDs among children in order to inform the development of more effective public health strategies.

The Global Burden of Disease (GBD) 2021 (7) provides a valuable tool for epidemiological research by integrating multidimensional health data at global, regional and national levels. It not only quantifies the burden of disease, but also provides a newer and more comprehensive assessment of health inequalities. The core of this approach is the Sociodemographic Index (SDI), which stratifies regions based on a combination of development indicators such as education, income and fertility. This stratification helps to identify absolute gaps and relative inequalities, revealing the complex relationship between socioeconomic development, temporal evolution and disease burden (10). The World Health Organization (WHO) has proposed a universal health coverage policy aimed at decreasing inequalities and achieving “health for all” (11). Emphasizing the inequalities of diseases across different regions and countries is crucial for optimizing resource allocation. However, there remains a lack of comprehensive analysis investigating global and regional cross-country inequalities in the burden of NDDs.

Here we describe the disease burden of three common NDDs: ASD, ADHD and IDID. We further conduct a cross-national inequality analysis based on the standard health equity analysis method recommended by the WHO. The results show that there are inequalities in the disease burden of these three common NDDs related to the level of sociodemographic development. We subsequently examined the magnitude of these inequalities and their temporal trends over the study period. We aim to provide references for the development of targeted prevention and treatment strategies, promote the effective allocation of resources, and ultimately improve global health outcomes.

## 2 Materials and methods

### 2.1 Data source

The GBD 2021 provides a comprehensive analysis of health impacts linked to 369 diseases, injuries, and conditions, along with 88 risk factors, covering 204 countries and territories. All data were obtained from publicly available databases following rigorous quality screening procedures. The GBD database incorporates published epidemiological studies relevant to disease burden, while excluding studies deemed incomplete, of low quality, or inconsistent with the study’s objectives (7). In this study, estimates and their 95% uncertainty intervals (UI) for the prevalence of ASD, ADHD, and IDID were obtained from the GBD 2021 dataset. The prevalence rates were expressed per 100,000 people. Additionally, the SDI—a composite index (0–1) reflecting income per capita, educational attainment, and fertility—was used to assess the sociodemographic development of countries and territories. According to GBD 2021, regions are grouped into five quintiles: low (0.00–0.4658), low-middle (0.4658–0.6188), middle (0.6188–0.7120), high-middle (0.7120–0.8103), and high (0.8103–1.00) (12). The University of Washington Institutional Review Board has granted a waiver of informed consent for the use of de-identified data in the GBD study.

### 2.2 Descriptive analysis

To comprehensively understand the burden of NDDs in children, descriptive analyses were conducted at global, regional, and country levels. The prevalence of NDDs among children worldwide from 1990 to 2021 was visually depicted and categorized by sex, age, and SDI quintiles. This approach offered insights into temporal trends and highlighted variations across different developmental contexts.

### 2.3 Joinpoint regression analysis

In this study, we used joinpoint regression to analyze the average annual percentage change (AAPC) and its corresponding 95% confidence interval (CI) in the prevalence of three common NDDs across 21 GBD regions, five SDI-level groups, and 204 countries from 1990 to 2021. Joinpoint is a robust statistical tool widely used in epidemiological research to assess temporal trends in disease prevalence and mortality. This methodology effectively segments the overall trend into distinct phases at identified inflection points, allowing for detailed assessment of trend dynamics over time. Based on the officially recommended joinpoint number, we set the maximum number of joinpoints to 6 (13). Each segment’s annual percent change (APC) and its 95% CI were calculated to quantify the magnitude of the epidemiological trends within specific intervals. Furthermore, we computed the AAPC for the periods 1990–2021, which involves weighting the regression coefficients of each segment by the duration of the segmented intervals. The AAPC and its 95% CI were also determined using the Monte Carlo permutation method with 4,499 randomly permuted datasets, ensuring robust statistical inference while maintaining the overall asymptotic significance level through Bonferroni correction (14). If both the APC/AAPC estimation and its lower boundary of the 95% CI were greater than zero, an increasing trend was identified for that period. Conversely, if both the APC/AAPC estimation and its upper boundary of the 95% CI were less than zero, a decreasing trend was noted. Otherwise, the trend was considered stable. This approach provides a comprehensive view of the changing epidemiological landscape of the disease under study.

### 2.4 Cross-country inequality analysis

The Slope Inequality Index (SII) and Concentration Index were used to quantify disparities in the prevalence of NDDs at global, regional, and national levels. The SII measures absolute inequality in a health indicator by assessing the difference in prevalence between the most and least advantaged subgroups within a population. This is achieved through a weighted regression model that incorporates the entire distribution of a socioeconomic determinant, such as education or wealth. The Concentration Index quantifies relative inequality, reflecting the extent to which a health indicator is disproportionately concentrated among socioeconomically advantaged or disadvantaged groups (15). To estimate absolute inequality, the SII was derived by regressing NDDs prevalence against a relative position scale based on the SDI. To account for heteroscedasticity, robust linear regression models were utilized. Specifically, iteratively reweighted least squares were applied, assigning smaller weights to observations with larger residuals, thereby mitigating the influence of outliers and ensuring more stable and reliable trend estimations (16). The Concentration Index was calculated using numerical integration of the Lorenz curve, which plots the cumulative proportion of NDD prevalence against the cumulative population distribution ranked by SDI. This approach provides a measure of relative inequality (17).

### 2.5 Predictive analysis

A log-linear age-period-cohort model was employed to forecast global prevalence rates and the number of prevalent cases from 2022 to 2046, based on recent trends. The NORDPRED software package, developed and implemented in R, has demonstrated strong empirical performance in projecting recent trends into the future (18). This model extrapolates the most recent 5-year period of observed data using a power function to stabilize growth trends. The linear trend observed in the most recent decade is then adjusted for the subsequent prediction periods (second, third, and fourth) by 25, 50, and 75%, respectively, either attenuating or accentuating the trend.

### 2.6 Statistics

The prevalence rate was expressed as the estimate per 100,000 population and its 95% UI. All analyses and visualization were executed using the Health Equity Assessment Toolkit from WHO and R software (V.4.4.1).

## 3 Results

### 3.1 Descriptive analysis the prevalence of NDDs

#### 3.1.1 Global level

In 2021, the global prevalence of ASD among children aged 0–14 was estimated at 857.14 per 100,000 (95% UI: 723.16 to 1009.04), while ADHD had a prevalence of 1661.61 per 100,000 (95% UI: 1128.43 to 2414.83), and IDID were reported at 1626.3 per 100,000 (95% UI: 908.58 to 2319.99). Regarding gender distribution, ASD prevalence in males was significantly higher at 1129.59 per 100,000 (95% UI: 953.43 to 1322.63) compared to females at 566.66 per 100,000 (95% UI: 475.15 to 674.81). Similarly, ADHD prevalence was markedly higher in males, reaching 2355.02 per 100,000 (95% UI: 1593.72 to 3401.9), compared to 922.3 per 100,000 (95% UI: 621.53 to 1363.21) in females. Conversely, IDID prevalence was slightly lower in males (1596.02 per 100,000; 95% UI: 840.01 to 2328.2) than in females (1658.59 per 100,000; 95% UI: 981.92 to 2310.72). In terms of age distribution, the prevalence of ASD and IDID remained relatively consistent across different pediatric age groups, whereas ADHD was more prevalent among children aged 5–9 and 10–14 years. Additionally, from the SDI perspective, ASD and ADHD were predominantly observed in higher SDI regions, while IDID cases were more concentrated in lower SDI regions.

The AAPC for the prevalence of three common NDDs among children worldwide from 1990 to 2021 were as follows: ASD increased at a rate of 0.09 (95% CI: 0.08 to 0.09), whereas ADHD and IDID exhibited declining trends, with AAPC of −0.08 (95% CI: −0.12 to −0.04) and −0.86 (95% CI: −0.88 to −0.84), respectively. Between 1990 and 2021, the prevalence of ASD increased in both boys (AAPC: 0.07, 95% CI: 0.06 to 0.07) and girls (AAPC: 0.11, 95% CI: 0.10 to 0.11). Conversely, ADHD prevalence declined in both boys (AAPC: −0.08, 95% CI: −0.13 to −0.03) and girls (AAPC: −0.10, 95% CI: −0.12 to −0.08). Similarly, IDID prevalence demonstrated a consistent downward trend in boys (AAPC: −0.91, 95% CI: −0.96 to −0.86) and girls (AAPC: −0.79, 95% CI: −0.80 to −0.77). Age-stratified analyses revealed that the AAPC for ASD were relatively stable across different age groups, clustering around 0.10. The most pronounced decline in ADHD prevalence was observed in children aged 10–14 years (AAPC: −0.28, 95% CI: −0.32 to −0.24). IDID prevalence exhibited the most significant reductions in children <5 years old (AAPC: −0.99, 95% CI: −1.03 to −0.95) and those aged 5–9 years (AAPC: −0.93, 95% CI: −0.96 to −0.89). When stratified by the SDI, ASD prevalence changes were most notable in middle-SDI regions (AAPC: 0.10, 95% CI: 0.09 to 0.10). ADHD prevalence showed the greatest increase in high-middle SDI regions (AAPC: 0.49, 95% CI: 0.42 to 0.55). In contrast, the most substantial declines in IDID prevalence were concentrated in low-middle SDI regions (AAPC: −1.32, 95% CI: −1.35 to −1.30) (Table 1).

**Table 1:** Prevalence rate of NDDs and their AAPC from 1990 to 2021 among children aged 0–14 years at the global level, stratified by sex, age, and SDI.

Characteristic	ASD				ADHD				IDID			
	1990	2021			1990	2021			1990	2021		
	Prevalence rate per 100,000 (95% UI)	Prevalence rate per 100,000 (95% UI)	AAPC, 1990–2021 (95% CI)	*p-*value	Prevalence rate per 100,000 (95% UI)	Prevalence rate per 100,000 (95% UI)	AAPC, 1990–2021 (95% CI)	*P-*value	Prevalence rate per 100,000 (95% UI)	Prevalence rate per 100,000 (95% UI)	AAPC, 1990–2021 (95% CI)	*P-*value
Global	834.79 (701.29 to 986.53)	857.14 (723.16 to 1009.04)	0.09 (0.08 to 0.09)	<0.001	1699.95 (1161.31 to 2483.73)	1661.61 (1128.43 to 2414.83)	−0.08 (−0.12 to −0.04)	<0.001	2120.66 (1211.42 to 2981.19)	1626.3 (908.58 to 2319.99)	-0.86 (−0.88 to −0.84)	<0.001
Sex												
Female	548.09 (458.57 to 650.85)	566.66 (475.15 to 674.81)	0.11 (0.1 to 0.11)	<0.001	950.57 (645.83 to 1384.66)	922.3 (621.53 to 1363.21)	-0.1 (−0.12 to −0.08)	<0.001	2116.22 (1271.97 to 2915.18)	1658.59 (981.92 to 2310.72)	-0.79 (−0.8 to −0.77)	<0.001
Male	1106.15 (930.68 to 1301.64)	1129.59 (953.43 to 1322.63)	0.07 (0.06 to 0.07)	<0.001	2409.2 (1652.77 to 3521.1)	2355.02 (1593.72 to 3401.9)	-0.08 (−0.13 to −0.03)	0.0011	2124.85 (1156.22 to 3043.68)	1596.02 (840.01 to 2328.2)	−0.91 (−0.96 to −0.86)	<0.001
Age, y												
<5	851.86 (714.87 to 1008.19)	877.73 (739.39 to 1034.74)	0.10 (0.08 to 0.11)	<0.001	199.28 (134.45 to 294.53)	194.77 (130.33 to 284.55)	−0.07 (−0.1 to −0.04)	<0.001	2146.31 (1230.49 to 3048.8)	1577.47 (864.68 to 2289.05)	−0.99 (−1.03 to −0.95)	<0.001
5–9	830.92 (698.13 to 980.77)	853.14 (719.38 to 1003.43)	0.09 (0.08 to 0.09)	<0.001	2141.28 (1458.39 to 3145.03)	2046.3 (1383.28 to 2985.89)	−0.15 (−0.2 to −0.11)	<0.001	2211.78 (1280.15 to 3080.9)	1654.66 (930.5 to 2341.57)	−0.93 (−0.96 to −0.89)	<0.001
10–14	819.26 (690.34 to 967.74)	840.95 (709.37 to 987.31)	0.08 (0.08 to 0.09)	<0.001	2955.9 (2036.48 to 4269.15)	2713.36 (1858.21 to 3908.68)	−0.28 (−0.32 to −0.24)	<0.001	1991.71 (1121.1 to 2803.42)	1645.28 (929.32 to 2332.43)	−0.61 (−0.63 to −0.59)	<0.001
SDI												
High	1118.7 (943.31 to 1309.6)	1,118 (940 to 1317.06)	0.00 (−0.02 to 0.01)	0.7331	2437.55 (1629.21 to 3545.85)	2722.67 (1812.53 to 3924.3)	0.36 (0.33 to 0.38)	<0.001	435.3 (87.76 to 823.79)	351.86 (62.7 to 683.5)	−0.69 (−0.72 to −0.66)	<0.001
High-middle	841.96 (705.18 to 996.18)	859.49 (721.26 to 1013.32)	0.07 (0.06 to 0.08)	<0.001	2296.17 (1586.65 to 3369.33)	2673.82 (1841.87 to 3876.26)	0.49 (0.42 to 0.55)	<0.001	703.07 (269.01 to 1143.14)	527.24 (171.73 to 887.45)	−0.92 (−0.96 to −0.89)	<0.001
Middle	749.89 (629.26 to 889.27)	772.26 (647.1 to 910.35)	0.10 (0.09 to 0.1)	<0.001	2038.67 (1402.33 to 2978.73)	2154.89 (1477.22 to 3134.88)	0.17 (0.12 to 0.22)	<0.001	1684.76 (957.23 to 2382.07)	1206.51 (640.05 to 1755.59)	−1.07 (−1.09 to −1.04)	<0.001
Low-middle	787.57 (662.96 to 932.62)	807.44 (679.76 to 947.92)	0.08 (0.08 to 0.08)	<0.001	1092.08 (731.3 to 1587.99)	1118.19 (748.98 to 1653.12)	0.07 (0.05 to 0.09)	<0.001	3917.15 (2421.55 to 5366.3)	2591.54 (1574.76 to 3564.73)	−1.32 (−1.35 to −1.3)	<0.001
Low	907.11 (762.34 to 1069.44)	925.42 (777.2 to 1091.29)	0.06 (0.06 to 0.07)	<0.001	784.75 (521.87 to 1158.4)	831.26 (553.54 to 1222.68)	0.19 (0.17 to 0.21)	<0.001	2584.86 (1486.68 to 3618.14)	1959.07 (1083.55 to 2800.94)	−0.89 (−0.91 to −0.87)	<0.001

NDDs, neurodevelopmental disorders; SDI, sociodemographic index; AAPC, average annual percentage change; ASD, autism spectrum disorders; ADHD, attention-deficit/hyperactivity disorder; IDID, Idiopathic developmental intellectual disability; y, years; CI, confidence interval; UI, uncertainty intervals.

#### 3.1.2 Regional level

In 2021, among the 21 GBD regions, the highest prevalence of ASD in boys was observed in the High-income Asia Pacific region, with a rate of 2271.52 (95% UI: 1915.80 to 2671.02) per 100,000 population. Similarly, the highest ASD prevalence in girls was also recorded in the same region, at 991.78 (95% UI: 833.81 to 1166.57) per 100,000. In contrast, the lowest ASD prevalence among boys was reported in Tropical Latin America, with a rate of 891.89 (95% UI: 750.67 to 1056.88) per 100,000, while the lowest prevalence among girls was found in East Asia, at 344.69 (95% UI: 283.19 to 415.83) per 100,000.

Regarding ADHD, the highest prevalence rates for both boys and girls were concentrated in Australia, with boys exhibiting a prevalence of 7266.56 (95% UI: 5207.71 to 9593.61) per 100,000 and girls at 2696.40 (95% UI: 1848.89 to 3779.00) per 100,000. Conversely, the lowest ADHD prevalence rates were observed in Central Sub-Saharan Africa, with boys at 1037.63 (95% UI: 683.36 to 1533.49) per 100,000 and girls at 428.91 (95% UI: 277.77 to 652.97) per 100,000.

**Figure 1:**
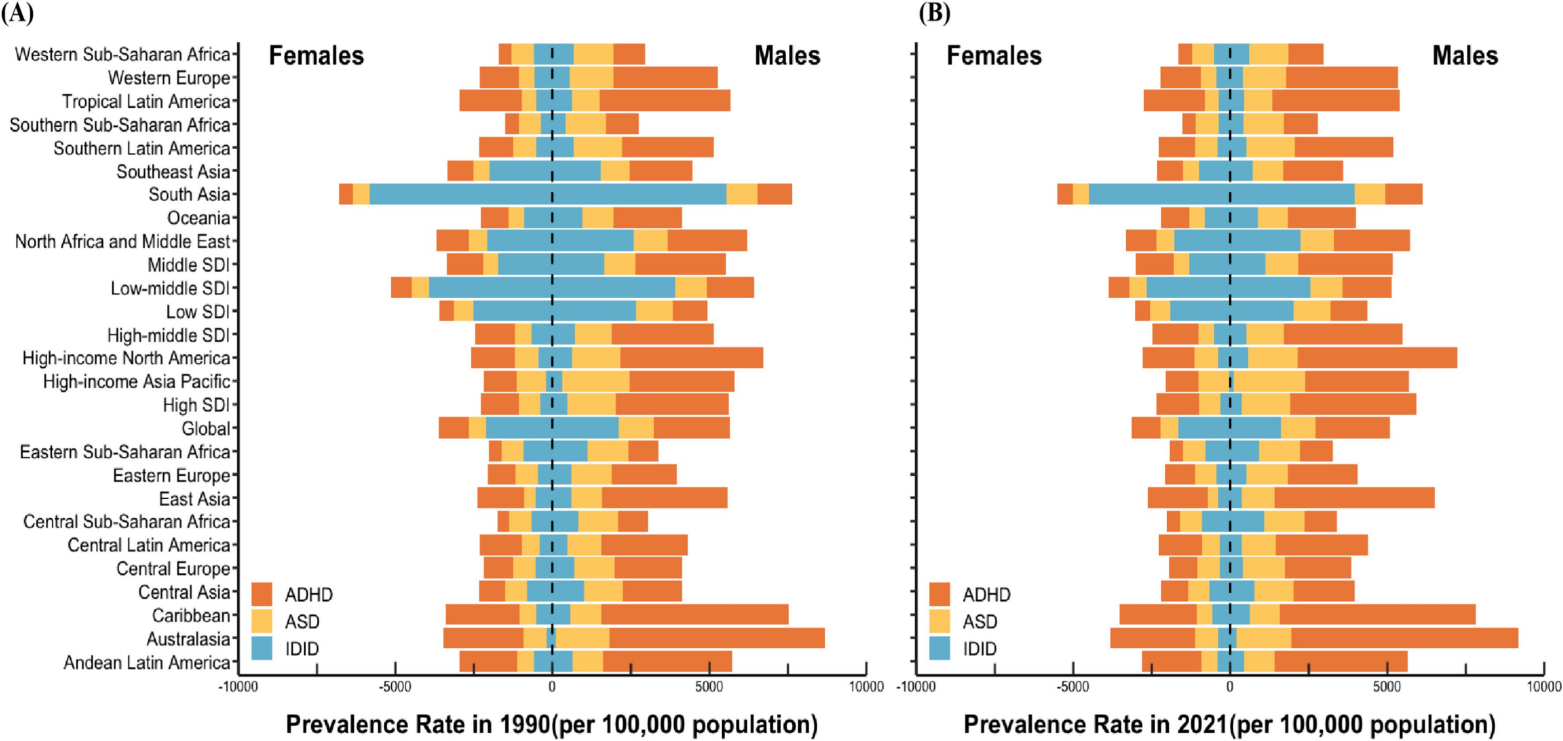
Prevalence rate of NDDs among children aged 0–14 in the world, various SDI regions, and 21 GBD regions in 1990 **(A)** and 2021 **(B)**. NDDs, neurodevelopmental disorders; SDI, sociodemographic index; GBD, Global Burden of Disease; ASD, autism spectrum disorders; ADHD, attention-deficit/hyperactivity disorder; IDID, Idiopathic developmental intellectual disability.

For IDID, the highest prevalence rates were recorded in South Asia, reaching 3959.46 (95% UI: 2449.13 to 5410.52) per 100,000 in boys and 4495.68 (95% UI: 3007.65 to 5957.18) per 100,000 in girls. In contrast, the lowest prevalence rates were observed in the High-income Asia Pacific region, where boys had a prevalence of 122.21 (95% UI: 5.48 to 394.74) per 100,000, and girls had a rate of 26.62 (95% UI: 0 to 110.89) per 100,000. It is noteworthy that this geographical distribution largely mirrors the pattern observed in 1990. However, a key difference is that in 1990, the region with the lowest IDID prevalence among girls aged 0–14 years was Australasia, whereas in 2021, this shifted to the High-income Asia Pacific region (Figure 1).

#### 3.1.3 National level

In 2021, among the 204 countries analyzed, Japan exhibited the highest prevalence of ASD among children aged 0–14 years, with an estimated rate of 1,678.36 per 100,000 (95% UI: 1,417.87 to 1,974.93). Conversely, Bangladesh recorded the lowest prevalence, at 652.14 per 100,000 (95% UI: 541.34 to 771.66). For ADHD, Australia had the highest prevalence, reaching 5,295.67 per 100,000 (95% UI: 3,822.54 to 7,027.16), while the United Arab Emirates reported the lowest prevalence, at 662.07 per 100,000 (95% UI: 428.80 to 972.40). Regarding IDID, India had the highest prevalence, at 4,951.66 per 100,000 (95% UI: 3,237.22 to 6,645.91), whereas Singapore reported the lowest prevalence, with an estimated rate of 17.49 per 100,000 (95% UI: 0 to 119.63).

**Figure 2:**
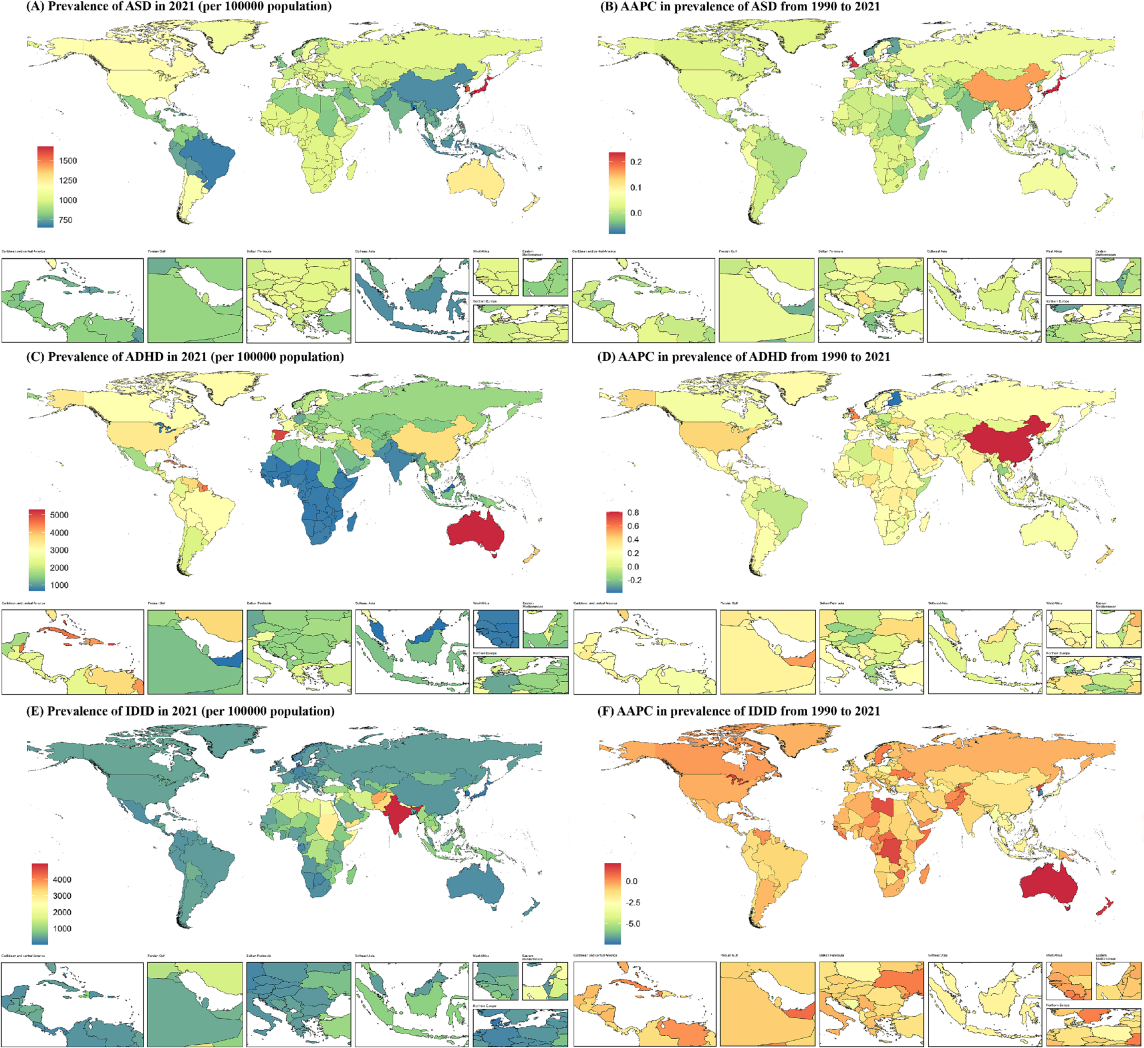
Prevalence of NDDs among children aged 0–14 at the national level in 2021 **(A,C,E)**, and their changing trends from 1990 to 2021 **(B,D,F)**. NDDs, neurodevelopmental disorders; AAPC, average annual percentage change; ASD, autism spectrum disorders; ADHD, attention-deficit/hyperactivity disorder; IDID, Idiopathic developmental intellectual disability.

The AAPC in the prevalence of the three common NDDs among children aged 0–14 years across 204 countries from 1990 to 2021 are summarized as follows: The highest increase in ASD prevalence was observed in Japan, with an AAPC of 0.24 (95% CI: 0.22 to 0.25), whereas the most significant decline was noted in Monaco, with an AAPC of −0.08 (95% CI: −0.11 to −0.06). ADHD exhibited the most rapid growth in China, with an AAPC of 0.81 (95% CI: 0.72 to 0.89), while Finland demonstrated the steepest decline, with an AAPC of −0.39 (95% CI: −0.50 to −0.28). The prevalence of IDID showed the highest increase in Australia, with an AAPC of 2.12 (95% CI: 2.01 to 2.23), whereas Singapore experienced the most substantial decrease, with an AAPC of −7.47 (95% CI: −7.98 to −6.96) (Figure 2).

### 3.2 Joinpoint regression analysis

**Figure 3:**
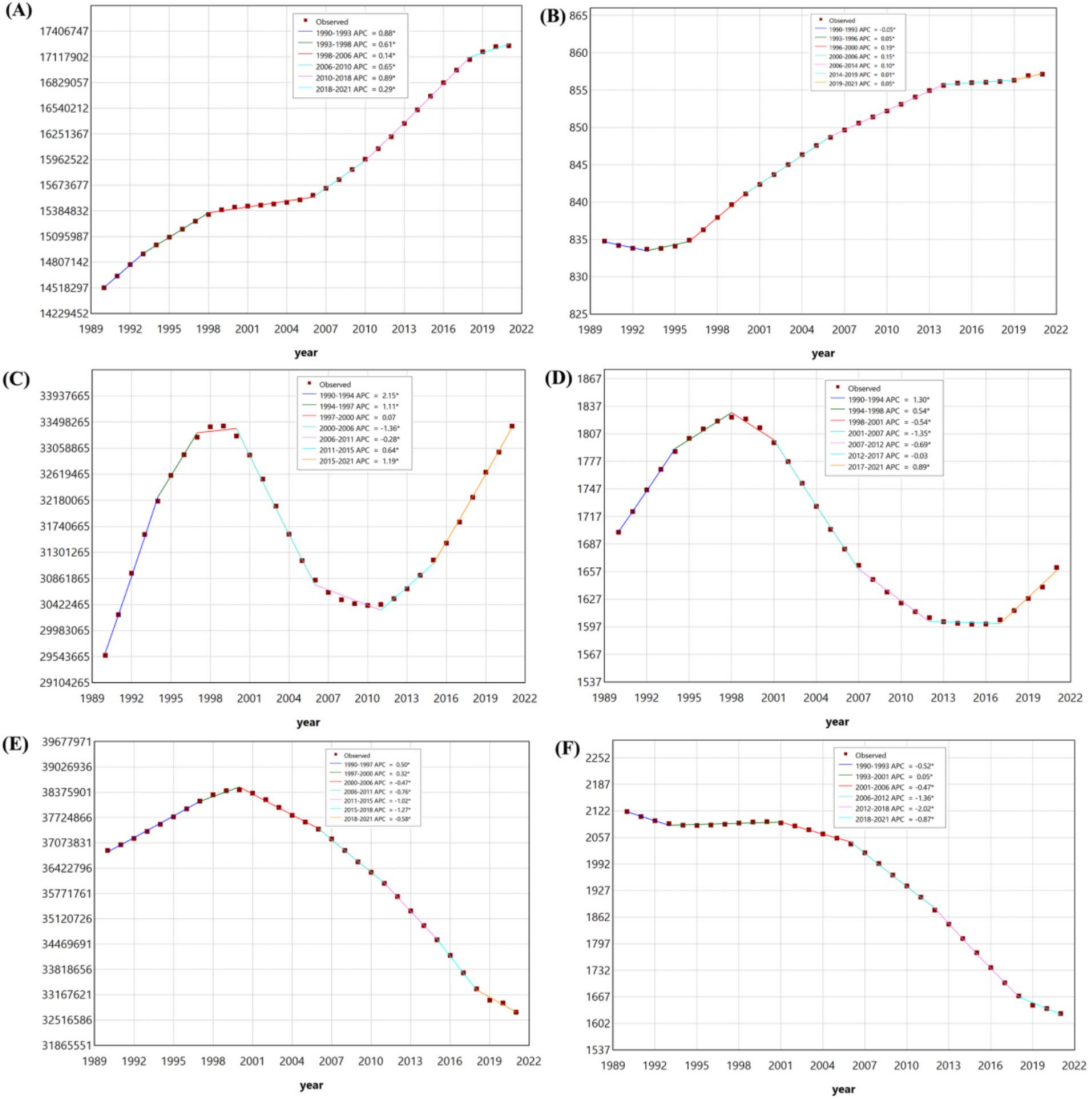
The joinpoint regression analysis on the case number of prevalent among children aged 0–14 in ASD **(A)**, ADHD **(C)**, and IDID **(E)**. The joinpoint regression analysis on the prevalence rate among children aged 0–14 in ASD **(B)**, ADHD **(D)**, and IDID **(F)**. ASD, autism spectrum disorders; ADHD, attention-deficit/hyperactivity disorder; IDID, Idiopathic developmental intellectual disability.

Figure 3 illustrates the results of joinpoint regression analyses for the temporal trends in prevalence and the number of prevalent cases of three common NDDs—ASD, ADHD, and IDID—among children aged 0–14 globally from 1990 to 2021.

#### 3.2.1 Trends in prevalence rate

Between 1990 and 2021, the prevalence rate of IDID demonstrated an overall downward trend. However, temporal fluctuations were evident across segmented periods. Notable declines were observed during five intervals: 1990–1993, 2001–2006, 2006–2012, 2012–2018, and 2018–2021. A modest increase occurred between 1993 and 2001. Among the periods of decline, the most pronounced reductions were detected in 2006–2012 and 2012–2018.

In contrast, the prevalence rate of ADHD exhibited a nonlinear pattern characterized by alternating periods of increase and decrease. Increases were identified during 1990–1994, 1994–1998, and 2017–2021, while decreases occurred during 1998–2001, 2001–2007, 2007–2012, and 2012–2017.

The prevalence rate of ASD showed a consistent upward trajectory across most of the study period. Specifically, consecutive increases were detected during six times intervals: 1993–1996, 1996–2000, 2000–2006, 2006–2014, 2014–2019, and 2019–2021. A slight decline was observed only during 1990–1993.

#### 3.2.2 Trends in the case number of prevalent

Regarding the case number of prevalent, IDID initially showed an upward trend from 1990 to 2000, followed by a gradual decline thereafter. The increases occurred during 1990–1997 and 1997–2000, while sustained reductions were observed during 2000–2006, 2006–2011, 2011–2015, 2015–2018, and 2018–2021.

The temporal pattern for ADHD was similarly dynamic. Increases in case numbers were noted in 1990–1994, 1994–1997, 1997–2000, 2011–2015, and 2015–2021, while decreases were observed during 2000–2006 and 2006–2011.

For ASD, the case number of prevalent increased steadily throughout the entire study period. Significant upward trends were identified during six successive intervals: 1990–1993, 1993–1998, 1998–2006, 2006–2010, 2010–2018, and 2018–2021.

### 3.3 Cross-country inequality analysis

**Figure 4:**
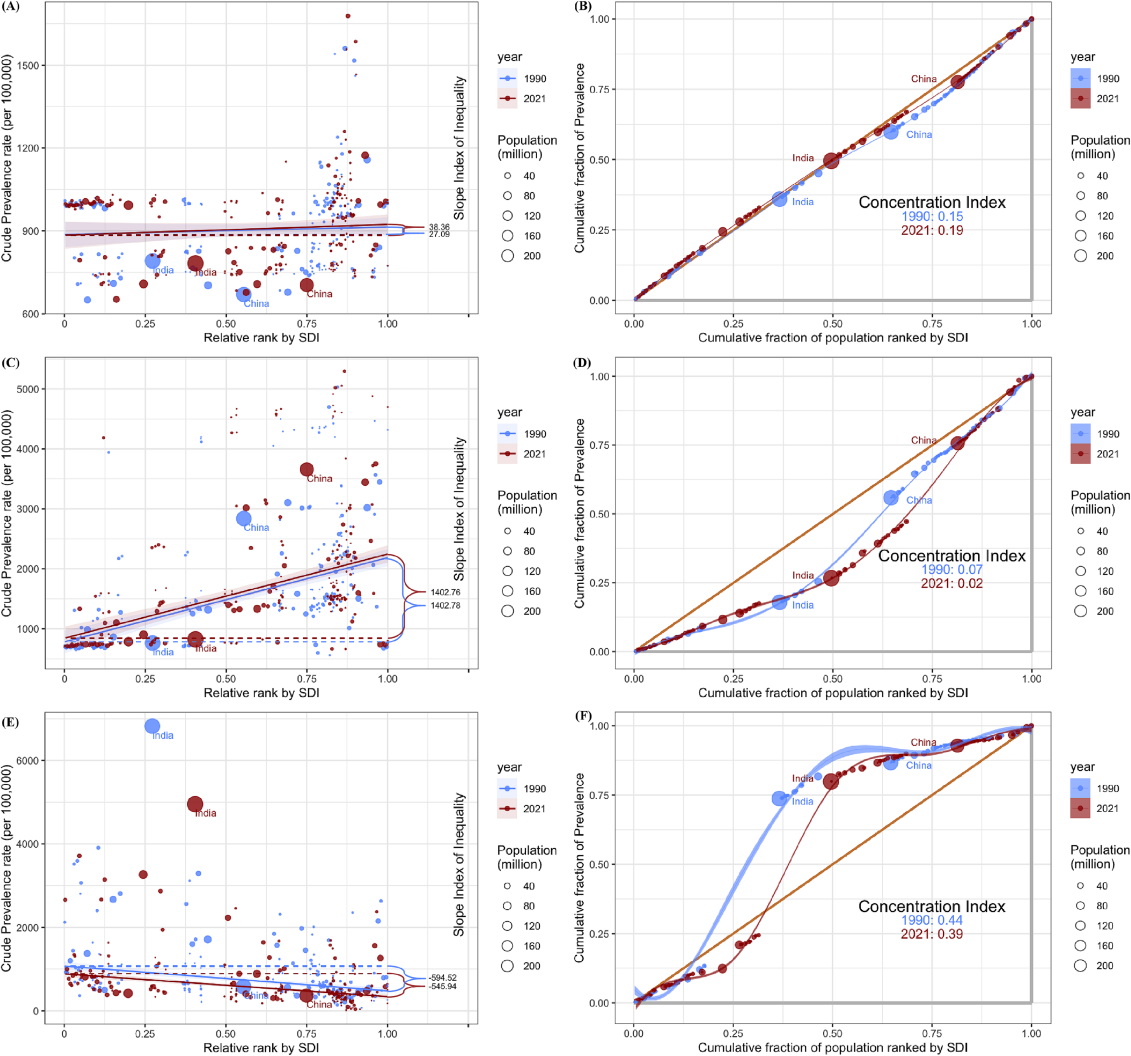
Health inequality regression curves and concentration curves for the prevalence among children aged 0–14 of ASD **(A,B)**, ADHD **(C,D)**, and IDID **(E,F)** worldwide, 1990 and 2021. ASD, autism spectrum disorders; ADHD, attention-deficit/hyperactivity disorder; IDID, Idiopathic developmental intellectual disability; SDI, Sociodemographic Index.

The SII (95% CI) changed from 27.09 (−29.98 to 84.17) in 1990 to 38.36 (−21.48 to 98.20) in 2021 for ASD, from 1402.78 (1100.25 to 1705.31) in 1990 to 1402.76 (1083.55 to 1721.97) in 2021 for ADHD, from −594.52 (−755.05 to −434.00) in 1990 to −545.94 (−673.19 to −418.69) in 2021 for IDID. Moreover, the concentration index(95% CI) showed 0.15 (0.07 to 0.23) in 1990 and 0.19 (0.10 to 0.26) in 2021 for ASD, 0.07 (−0.02 to 0.16) in 1990 and 0.02 (−0.07 to 0.11) in 2021 for ADHD, 0.44 (0.34 to 0.53) in 1990 and 0.39 (0.28 to 0.48) in 2021 for IDID (Figure 4).

### 3.4 Predictive analysis

It is estimated that by 2046, the number of children aged 0–14 with ASD will reach 15,823,257 (5,080,091 girls and 10,743,166 boys), the number of children with ADHD will reach 29,867,591 (8,084,274 girls and 21,783,317 boys), and the number of children with IDID will reach 32,056,358 (15,872,619 girls and 16,183,739 boys). In general, the number of children aged 0–14 with the three common NDDs in 2046 will decrease compared with 2021.

**Figure 5:**
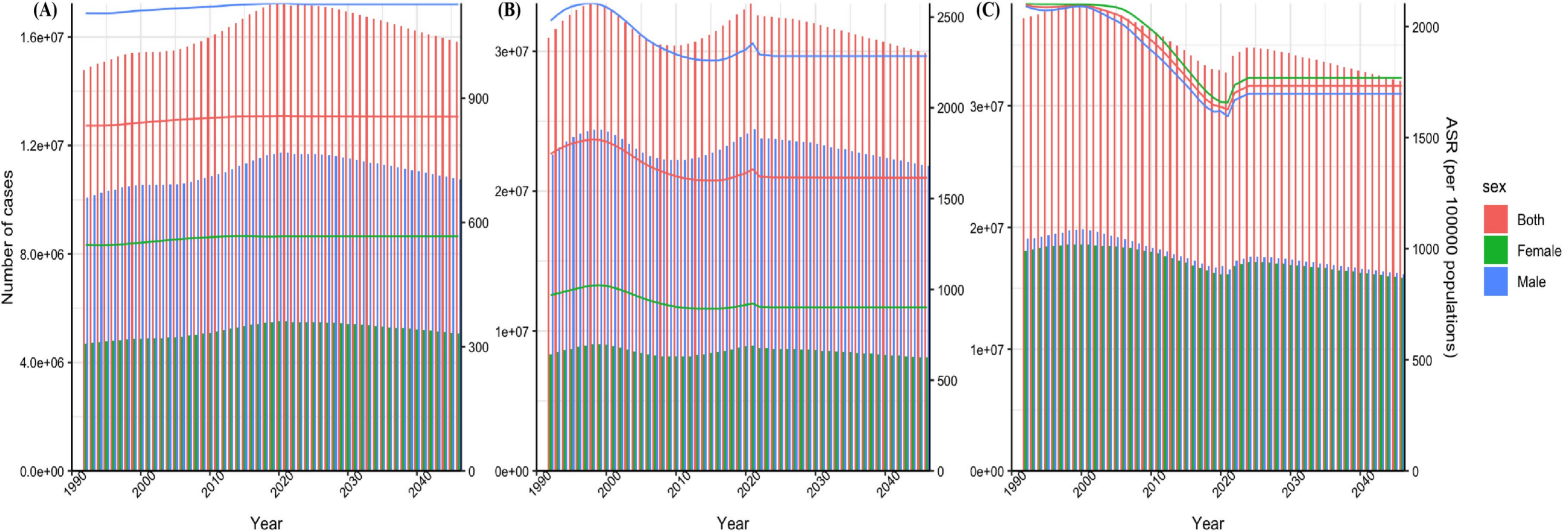
Prediction of disease burden for NDDs by 2046. Nordpred method predicted results of ASPR among children aged 0–14 in ASD **(A)**, ADHD **(B)**, and IDID **(C)**. NDDs, neurodevelopmental disorders; ASD, autism spectrum disorders; ADHD, attention-deficit/hyperactivity disorder; IDID, idiopathic developmental intellectual disability; ASR, age-standardized rate; ASPR, age-standardized prevalence rates.

By 2046, the age-standardized prevalence rates (ASPR) per 100,000 population among children aged 0–14 with ASD, ADHD, and IDID will be 855.28 (764.64 to 955.88), 1614.40 (1240.40 to 2145.35), and 1732.71 (1330.81 to 2130.14). The ASPR of ASD and ADHD in 2046 decreased slightly compared with 2021, while the results of IDID showed an increase. The years with faster increases were concentrated in 2022, 2023 and 2024, among which the fastest increase was in 2022 (Figure 5).

## 4 Discussion

Neurodevelopmental disorders pose a significant global public health challenge, with their associated disease burden drawing considerable research attention. This study is the first to comprehensively analyze the burden of three common NDDs (ASD, ADHD, and IDID) among children aged 0–14 in recent years. This study analyzes temporal trends and cross-country inequalities in NDDs among children aged 0–14 over the past 30 years and projects the disease burden for the next 25 years, based on data from the GBD 2021 study.

The prevalence trends of the three common NDDs in children aged 0–14 from 1990 to 2021 varied significantly. For ASD, one contributing factor to the rising prevalence is the expansion of diagnostic categories in the DSM-IV, which included additional autism-related subtypes such as childhood disintegrative disorder, Rett’s disorder, and pervasive developmental disorder not otherwise specified (PDD-NOS) under the umbrella of pervasive developmental disorders. Furthermore, the DSM-5 later introduced the concept of spectrum disorders, consolidating previously separate diagnoses—including autistic disorder, childhood disintegrative disorder, Asperger’s syndrome, and PDD-NOS—into a single diagnostic entity (19). Research indicates that changes in diagnostic criteria alone account for approximately 30% of the observed growth in ASD diagnoses. Furthermore, the adoption of the Modified Checklist for Autism in Toddlers (M-CHAT) screening tool by the WHO in 2007 significantly enhanced early detection. The ASD screening rate at 18 months of age increased from 40 to 75%, underscoring the impact of improved diagnostic capabilities on prevalence data (20). Additionally, demographic shifts, particularly the rising proportion of older parents, have contributed to increased ASD risk (21). Studies show that paternal age over 40 is associated with a 1.5-fold increase in ASD risk (22). Environmental factors also play a crucial role. Increasing levels of air pollution, particularly exposure to fine particulate matter (PM2.5), have been identified as a dose-dependent risk factor for ASD (23). This highlights the importance of both genetic predisposition and environmental influences in ASD etiology.

The global prevalence of ADHD among children exhibited a fluctuating trend between 1990 and 2021, characterized by an initial rise from 1990 to 1998, a decline from 1998 to 2017, and a subsequent resurgence from 2017 to 2021. The increase in ADHD prevalence observed between 1990 and 1998 may be partially explained by the revision of diagnostic criteria in the DSM-III-R (1987), which unified attention deficits and hyperactivity/impulsivity into a single diagnostic entity—ADHD—a term that has remained in clinical use. Additionally, the expansion of attention-deficit subtypes in the DSM-IV (1994), together with the widespread implementation of systematic school-based ADHD screening following the enactment of the Individuals with Disabilities Education Act in the United States in 1990, may have further contributed to this upward trend (24). The decline observed in the second phase may be attributed to changes in diagnostic practices. In particular, the revision of the American Academy of Pediatrics (AAP) guidelines in 2011 mandated that ADHD symptoms must be present in at least two different settings, which likely led to more stringent diagnostic criteria and a reduction in overdiagnosis (25). The renewed increase in ADHD prevalence in the third phase has been closely associated with the growing global use of electronic devices. Studies have demonstrated a significant correlation between prolonged exposure to digital media and ADHD symptomatology, highlighting the potential impact of modern screen time habits on attention regulation in children (26). Additionally, increasing awareness of ADHD among clinicians, teachers, and parents may be another factor contributing to the rising prevalence during this period, as greater recognition of the disorder has led to a sustained increase in reported diagnoses in recent years (27).

The global prevalence of IDID in children has shown a consistent downward trend from 1990 to 2021. This decline is attributed to several key factors, including the widespread implementation of neonatal screening technologies, folic acid fortification policies, and advancements in perinatal medical care. Since the 1990s, the global promotion of neonatal metabolic disease screening, such as for phenylketonuria (PKU) and congenital hypothyroidism has facilitated early diagnosis and treatment, significantly reducing preventable cases. Research data indicate that the neonatal PKU screening rate in the United States increased from 85% in 1990 to 99.8% in 2020, leading to a 72% reduction in related IDID prevalence (28). The mandatory fortification of grain products with folic acid, introduced in the United States in 1998, has played a crucial role in reducing the global incidence of neural tube defects (NTDs), subsequently lowering the number of secondary IDID cases. Folic acid deficiency is a leading cause of NTDs, and several children with NTDs develop IDID (29). Advancements in perinatal medical care, including fetal ultrasound monitoring and gestational diabetes management, have contributed to the reduced risk of birth asphyxia and preterm birth, which are both significant risk factors for IDID (30). These collective efforts highlight the significant progress made in reducing the burden of IDID through proactive public health policies, technological advancements, and improved medical interventions.

The ASD and ADHD are predominantly diagnosed in males, a trend largely attributed to biological inheritance and diagnostic bias. From a genetic perspective, several ASD-associated pathogenic genes, such as *NLGN3* and *NLGN4X*, are located on the X chromosome. Because males (XY) have only a single copy of the X chromosome, mutations in these genes can directly contribute to disease manifestation (31). Additionally, hormonal influences play a crucial role in the pathophysiology of ASD and ADHD. Studies suggest that elevated prenatal testosterone levels may increase susceptibility to these disorders in males by affecting neuronal migration and synaptic pruning, key processes in brain development (32). Moreover, sex-based differences in neurodevelopment have been observed. In particular, males exhibit stronger connectivity within the default mode network (DMN), a neural system implicated in self-referential thinking and social cognition. This heightened connectivity has been associated with the repetitive behavior characteristic of ASD as well as the impulsivity commonly seen in ADHD (33). In females, ADHD is more likely to manifest as inattentiveness rather than hyperactivity, which may lead to underdiagnosis or misdiagnosis. Similarly, females with autism spectrum disorder (ASD) often exhibit stronger social camouflage abilities, allowing them to mask their symptoms more effectively and potentially delaying recognition and intervention. Additionally, gender bias in screening tools plays a critical role in diagnostic discrepancies. Many ADHD assessment scales, such as the Conners Rating Scales, were primarily developed based on male symptomatology, making them less sensitive to detecting ADHD presentations in females (34). The distribution of diseases among children of different age groups varies, with ASD and IDID showing similar prevalence patterns across the three age groups: under 5 years, 5–9 years, and 10–14 years. In contrast, ADHD is predominantly observed in the 5–9 and 10–14 age groups, with a lower prevalence in children under 5 years old. This distribution can be attributed to differences in the onset and recognition of core symptoms. ASD is typically characterized by early manifestations such as language delay and social withdrawal, which often become apparent by 2–3 years of age (35). Many developed countries have implemented standardized screening protocols, such as the Modified Checklist for Autism in Toddlers (M-CHAT) at 18–24 months, leading to a higher diagnostic rate in children under 5 years old (36). Similarly, IDID is often caused by genetic factors or perinatal insults and can be identified early through developmental milestone assessments (37). In contrast, the core symptoms of ADHD, such as inattention and hyperactivity, are more likely to be overlooked in children under 5, as behavioral expectations at this age are less stringent (38). However, as children enter school, increasing academic and behavioral demands make these symptoms more noticeable, leading to a higher diagnostic rate in school-aged children (34). Additionally, the diagnostic criteria for ADHD require symptoms to be present before the age of 12, but in clinical practice, diagnosis is most commonly made during school years, contributing to the lower reported prevalence in children under 5 (34).

The variations in the distribution of these three diseases across different SDI regions can be attributed to several factors. Notably, the prevalence of ASD and ADHD is higher in high-SDI countries. One key reason is the disparity in diagnostic resources: comprehensive screening programs for ASD and ADHD are well-established in high-SDI regions, whereas low-SDI regions experience a significantly higher rate of missed diagnoses due to limited access to diagnostic services (39). In Japan, following the enactment of the Developmental Disabilities Support Act in 2005, ASD was formally integrated into the national social welfare system. This policy change facilitated access to educational subsidies upon diagnosis, leading to a threefold increase in the ASD diagnosis rate (40). In Bangladesh, the underreporting of health conditions may be influenced by several factors, including a shortage of medical specialists, limited healthcare services at the primary care level, and a prevailing preference for traditional medicine (41). In some developing countries with low SDI levels, cultural beliefs, disease-related stigma, and differing health perceptions may contribute to the underdiagnosis of ASD and ADHD (42). For instance, in certain Asian cultures, avoiding eye contact with authority figures is traditionally regarded as a sign of respect or modesty, whereas in some African and Latin American cultures, direct eye contact may be perceived as disrespectful (43). As a result, children who avoid eye contact with parents, teachers, or clinicians may be viewed as well-behaved rather than exhibiting a core symptom of ASD. Such cultural interpretations can delay or obscure the identification and diagnosis of ASD (44). At the same time, the stigma associated with the disease cannot be overlooked, as it directly contributes to a lower rate of medical consultation and treatment. This perspective is further supported by a study on the prevalence of ADHD in the United Arab Emirates (45). Moreover, advanced maternal age and increased exposure to electronic screens are more prevalent in high SDI regions. The potential implications of these factors on ASD and ADHD have been discussed earlier (46). The burden of IDID is predominantly concentrated in low- and low-middle SDI regions, where its prevalence remains high due to factors such as malnutrition (with maternal iodine deficiency increasing the risk of IDID by 2.5 times), infections (e.g., congenital rubella, meningitis), and inadequate prenatal care (47). Notably, Singapore recorded the lowest prevalence of IDID among 204 countries in 2021 and exhibited the most significant decline in IDID prevalence from 1990 to 2021. This remarkable achievement can be attributed to Singapore’s comprehensive three-tiered prevention system. It was among the first countries in the world to implement national legislation mandating folic acid fortification. Since then, the incidence of neural tube defect-related IDID has dropped significantly compared to before implementation (48). Meanwhile, the country is actively implementing expanded newborn screening using tandem mass spectrometry technology, enabling the detection of up to 50 metabolic disorders. Additionally, appropriate medical interventions have been established for identified cases to ensure timely treatment and management (49).

The results of the health inequality analysis in this study indicated that the SII for both ASD and ADHD was positive, suggesting that the burden of these two disorders is disproportionately higher in countries with higher sociodemographic development. Several factors may explain this phenomenon. First, high-SDI countries often possess greater diagnostic capacity, resulting in higher detection rates (38). Second, certain risk factors associated with these disorders, such as advanced parental age and prolonged exposure to electronic screens may be more prevalent in these settings (50, 51). For example, Australia reported the highest prevalence of ADHD among children aged 0–14 years globally. Several factors may account for this finding. Firstly, as a high-SDI country, Australia has a high level of child healthcare coverage and a relatively well-developed primary healthcare system, which ensures broad access to medical services for children (52). Secondly, the high level of public awareness regarding ADHD may contribute to increased recognition, assessment, and reporting of cases (53). Finally, in recent years, Australia has developed a national clinical practice guideline for ADHD, which was commissioned in 2018 with funding from the Australian Department of Health; during the development process, there has been a growing awareness and recognition of ADHD among both the public and professionals (4). In contrast, the comparatively lower burden observed in low-SDI countries may reflect underdiagnosis due to limited awareness, cultural stigma, and differences in health-seeking behavior, rather than a true lower prevalence (54).

The SII for IDID was negative, indicating that the burden of this disorder is more severe in countries with lower sociodemographic development. As previously discussed, this may be attributed to limited access to prenatal screening, suboptimal maternal and child nutrition, inadequate perinatal care, and insufficient infection control measures in low-SDI settings.

The epidemiological rationale for predicting the disease burden of ASD, ADHD, and IDID in children aged 0–14 from 2021 to 2046 is as follows: The slight decline in the standardized prevalence of ASD and ADHD compared to previous periods can be attributed to the stabilization of diagnostic criteria and the correction of overdiagnosis. Additionally, advancements in genetic screening and prenatal interventions have contributed to a reduction in genetically linked ASD cases (55). Improved regulation of digital media exposure has also played a role in mitigating the disease burden of ADHD. Furthermore, the widespread implementation of early intervention programs has alleviated certain clinical symptoms, preventing some children from meeting diagnostic thresholds. The more pronounced increase in the ASPR of IDID between 2022 and 2024 is likely associated with the delayed effects of the COVID-19 pandemic, which disrupted prenatal care and delayed newborn screening during this period. Projections of the number of children aged 0–14 diagnosed with these three conditions indicate an overall decline by 2046 compared to 2021. The primary driver of this trend is the continued global decline in fertility rates. Forecasts suggest that the global population of children aged 0–14 will decrease from 2.0 billion in 2021 to 1.8 billion in 2046 (56). It is important to acknowledge certain limitations in the forecasting component of this study. Specifically, potential changes in disease classification systems, diagnostic criteria, or the implementation of public health interventions over time may influence future trends and introduce uncertainty into the projections. Our estimates of disease burden are based solely on the extrapolation of current prevalence trends and anticipated demographic changes, including population aging and growth, and do not account for unforeseen policy shifts or advancements in diagnostic practices.

## 5 Conclusion

Differences in gender and age distribution among the three common NDDs in children result from the combined influence of biological susceptibility and sociocultural factors. In contrast, regional variations in prevalence primarily reflect disparities in public health resources. The relatively high prevalence of ASD and ADHD in high-SDI countries is primarily attributed to enhanced diagnostic capabilities, greater awareness among healthcare professionals and the general public, and better access to specialized services. In contrast, the lower reported prevalence in low-SDI countries may not reflect the true disease burden, as it may be influenced by cultural beliefs, stigma, and limited recognition or understanding of these conditions. Conversely, the high prevalence of IDID in low-SDI countries is more likely driven by factors such as malnutrition, untreated infections, and inadequate prenatal and perinatal care. Looking ahead, the underlying causes of the burden of NDDs, as well as their associations with socioeconomic status, environmental exposures, genetic predispositions, diagnostic criteria, and cultural factors, warrant further investigation to elucidate potential mechanisms and contributing pathways. The findings of this study may serve as a scientific foundation for informing public health policy, particularly in low-SDI regions. Strengthening investments in perinatal care, neonatal screening programs, and child mental health services, alongside improving healthcare infrastructure in resource-limited settings, is urgently needed. Moreover, enhancing public awareness and education about NDDs among parents, teachers, and the broader community is essential to reduce stigma, improve recognition of symptoms, and increase timely access to diagnosis and treatment.

## Data Availability

The datasets presented in this study can be found in online repositories. The names of the repository/repositories and accession number(s) can be found at: https://ghdx.healthdata.org/gbd-2021.

## References

[1] Thapar A Cooper M Rutter M. Neurodevelopmental disorders. Lancet Psychiatry. (2017) 4:339–46. doi: 10.1016/S2215-0366(16)30376-5, PMID: 27979720

[2] Harris JC. New classification for neurodevelopmental disorders in DSM-5. Curr Opin Psychiatry. (2014) 27:95–7. doi: 10.1097/YCO.0000000000000042, PMID: 24441422

[3] Joon P Kumar A Parle M. What is autism? Pharmacol Rep. (2021) 73:1255–64. doi: 10.1007/s43440-021-00244-0, PMID: 33694100

[4] May T Birch E Chaves K Cranswick N Culnane E Delaney J. The Australian evidence-based clinical practice guideline for attention deficit hyperactivity disorder. Aust N Z J Psychiatry. (2023) 57:1101–16. doi: 10.1177/00048674231166329, PMID: 37254562 PMC10363932

[5] Ordoñez-Razo RM Gutierrez-López Y Araujo-Solis MA Benitez-King G Ramírez-Sánchez I Galicia G. Overexpression of miR-25 downregulates the expression of ROBO2 in idiopathic intellectual disability. Int J Mol Sci. (2024) 25:3953. doi: 10.3390/ijms25073953, PMID: 38612763 PMC11011991

[6] Galasso C Lo-Castro A El-Malhany N Curatolo P. "idiopathic" mental retardation and new chromosomal abnormalities. Ital J Pediatr. (2010) 36:17. doi: 10.1186/1824-7288-36-17, PMID: 20152051 PMC2844383

[7] GBD 2021 Diseases and Injuries Collaborators. Global incidence, prevalence, years lived with disability (YLDs), disability-adjusted life-years (DALYs), and healthy life expectancy (HALE) for 371 diseases and injuries in 204 countries and territories and 811 subnational locations, 1990-2021: a systematic analysis for the global burden of disease study 2021. Lancet. (2024) 403:2133–61. doi: 10.1016/S0140-6736(24)00757-8, PMID: 38642570 PMC11122111

[8] Bourke-Taylor HM Lee DA Tirlea L Joyce K Morgan P Haines TP. Interventions to improve the mental health of mothers of children with a disability: systematic review, meta-analysis and description of interventions. J Autism Dev Disord. (2021) 51:3690–706. doi: 10.1007/s10803-020-04826-4, PMID: 33389452

[9] Gee DG. Neurodevelopmental mechanisms linking early experiences and mental health: translating science to promote well-being among youth. Am Psychol. (2022) 77:1033–45. doi: 10.1037/amp0001107, PMID: 36595400 PMC9875304

[10] Hosseinpoor AR Bergen N Schlotheuber A. Promoting health equity: WHO health inequality monitoring at global and national levels. Glob Health Action. (2015) 8:29034. doi: 10.3402/gha.v8.29034, PMID: 26387506 PMC4576419

[11] Verrecchia R Thompson R Yates R. Universal health coverage and public health: a truly sustainable approach. Lancet Public Health. (2019) 4:e10–1. doi: 10.1016/S2468-2667(18)30264-0, PMID: 30551975

[12] Xu C Jiang C Liu X Shi W Bai J Mubarik S. Epidemiological and sociodemographic transitions in the global burden and risk factors for Alzheimer's disease and other dementias: a secondary analysis of GBD 2021. Int J Equity Health. (2025) 24:149. doi: 10.1186/s12939-025-02530-2, PMID: 40413544 PMC12103806

[13] National Cancer Institute (2024). Joinpoint trend analysis software. Available online at: https://surveillance.cancer.gov/joinpoint/ (Accessed February 19, 2025).

[14] Kim HJ Fay MP Feuer EJ Midthune DN. Permutation tests for joinpoint regression with applications to cancer rates. Stat Med. (2000) 19:335–51. doi: 10.1002/(SICI)1097-0258(20000215)19:3<335::AID-SIM336>3.0.CO;2-Z, PMID: 10649300

[15] Howe LD. Handbook on health inequality monitoring. Int J Epidemiol. (2014) 43:1345–6. doi: 10.1093/ije/dyu124

[16] Rousseeuw PJ Hampel FR Ronchetti EM Stahel WA. Robust statistics: the approach based on influence functions. New York: Wiley (1986).

[17] Wagstaff A Paci P van Doorslaer E. On the measurement of inequalities in health. Soc Sci Med. (1991) 33:545–57. doi: 10.1016/0277-9536(91)90212-U, PMID: 1962226

[18] Møller B Fekjaer H Hakulinen T Sigvaldason H Storm HH Talbäck M. Prediction of cancer incidence in the Nordic countries: empirical comparison of different approaches. Stat Med. (2003) 22:2751–66. doi: 10.1002/sim.1481, PMID: 12939784

[19] Tsai LY Ghaziuddin M. DSM-5 ASD moves forward into the past. J Autism Dev Disord. (2014) 44:321–30. doi: 10.1007/s10803-013-1870-3, PMID: 23807202

[20] Johnson CP Myers SM. Identification and evaluation of children with autism spectrum disorders. Pediatrics. (2007) 120:1183–215. doi: 10.1542/peds.2007-2361, PMID: 17967920

[21] Timæus IM Moultrie TA. Pathways to low fertility: 50 years of limitation, curtailment, and postponement of childbearing. Demography. (2020) 57:267–96. doi: 10.1007/s13524-019-00848-5, PMID: 31970647 PMC7051933

[22] Sandin S Schendel D Magnusson P Hultman C Surén P Susser E. Autism risk associated with parental age and with increasing difference in age between the parents. Mol Psychiatry. (2016) 21:693–700. doi: 10.1038/mp.2015.70, PMID: 26055426 PMC5414073

[23] Becerra TA Wilhelm M Olsen J Cockburn M Ritz B. Ambient air pollution and autism in Los Angeles county, California. Environ Health Perspect. (2013) 121:380–6. doi: 10.1289/ehp.1205827, PMID: 23249813 PMC3621187

[24] Wolraich ML McKeown RE Visser SN Bard D Cuffe S Neas B. The prevalence of ADHD: its diagnosis and treatment in four school districts across two states. J Atten Disord. (2014) 18:563–75. doi: 10.1177/1087054712453169, PMID: 22956714

[25] Wolraich M Brown L Brown RT DuPaul G Earls M Feldman HM. ADHD: clinical practice guideline for the diagnosis, evaluation, and treatment of attention-deficit/hyperactivity disorder in children and adolescents. Pediatrics. (2011) 128:1007–22. doi: 10.1542/peds.2011-2654, PMID: 22003063 PMC4500647

[26] Liu H Chen X Huang M Yu X Gan Y Wang J. Screen time and childhood attention deficit hyperactivity disorder: a meta-analysis. Rev Environ Health. (2024) 39:643–50. doi: 10.1515/reveh-2022-0262, PMID: 37163581

[27] Abdelnour E Jansen MO Gold JA. ADHD diagnostic trends: increased recognition or overdiagnosis? Mo Med. (2022) 119:467–73. doi: 10.1001/jamanetworkopen.2018.1471, PMID: 36337990 PMC9616454

[28] McCandless SE Wright EJ. Mandatory newborn screening in the United States: history, current status, and existential challenges. Birth Defects Res. (2020) 112:350–66. doi: 10.1002/bdr2.1653, PMID: 32115905

[29] Paneth N. The contribution of epidemiology to the understanding of neurodevelopmental disabilities. Dev Med Child Neurol. (2023) 65:1551–6. doi: 10.1111/dmcn.15633, PMID: 37149891

[30] Chen S Wang X Lee BK Gardner RM. Associations between maternal metabolic conditions and neurodevelopmental conditions in offspring: the mediating effects of obstetric and neonatal complications. BMC Med. (2023) 21:422. doi: 10.1186/s12916-023-03116-x, PMID: 37936224 PMC10631144

[31] Werling DM Brand H An JY Stone MR Zhu L Glessner JT. An analytical framework for whole-genome sequence association studies and its implications for autism spectrum disorder. Nat Genet. (2018) 50:727–36. doi: 10.1038/s41588-018-0107-y, PMID: 29700473 PMC5961723

[32] Baron-Cohen S Auyeung B Nørgaard-Pedersen B Hougaard DM Abdallah MW Melgaard L. Elevated fetal steroidogenic activity in autism. Mol Psychiatry. (2015) 20:369–76. doi: 10.1038/mp.2014.48, PMID: 24888361 PMC4184868

[33] Satterthwaite TD Wolf DH Roalf DR Ruparel K Erus G Vandekar S. Linked sex differences in cognition and functional connectivity in youth. Cereb Cortex. (2015) 25:2383–94. doi: 10.1093/cercor/bhu036, PMID: 24646613 PMC4537416

[34] Wolraich ML Hagan JF Jr Allan C Chan E Davison D Earls M. Clinical practice guideline for the diagnosis, evaluation, and treatment of attention-deficit/hyperactivity disorder in children and adolescents. Pediatrics. (2019) 144:e20192528. doi: 10.1542/peds.2019-2528, PMID: 31570648 PMC7067282

[35] Zwaigenbaum L Bauman ML Choueiri R Fein D Kasari C Pierce K. Early identification and interventions for autism spectrum disorder: executive summary. Pediatrics. (2015) 136:S1–9. doi: 10.1542/peds.2014-3667B 26430167 PMC9923899

[36] Zwaigenbaum L Bauman ML Choueiri R Kasari C Carter A Granpeesheh D. Early intervention for children with autism Spectrum disorder under 3 years of age: recommendations for practice and research. Pediatrics. (2015) 136:S60–81. doi: 10.1542/peds.2014-3667E 26430170 PMC9923898

[37] Moeschler JB Shevell M. Comprehensive evaluation of the child with intellectual disability or global developmental delays. Pediatrics. (2014) 134:e903–18. doi: 10.1542/peds.2014-1839 25157020 PMC9923626

[38] Sayal K Prasad V Daley D Ford T Coghill D. Adhd in children and young people: prevalence, care pathways, and service provision. Lancet Psychiatry. (2018) 5:175–86. doi: 10.1016/S2215-0366(17)30167-0, PMID: 29033005

[39] Erskine HE Baxter AJ Patton G Moffitt TE Patel V Whiteford HA. The global coverage of prevalence data for mental disorders in children and adolescents. Epidemiol Psychiatr Sci. (2017) 26:395–402. doi: 10.1017/S2045796015001158, PMID: 26786507 PMC6998634

[40] Sasayama D Kuge R Toibana Y Honda H. Trends in autism Spectrum disorder diagnoses in Japan, 2009 to 2019. JAMA Netw Open. (2021) 4:e219234. doi: 10.1001/jamanetworkopen.2021.9234, PMID: 33944926 PMC8097493

[41] Hossain MD Ahmed HU Jalal Uddin MM Chowdhury WA Iqbal MS Kabir RI. Autism spectrum disorders (ASD) in South Asia: a systematic review. BMC Psychiatry. (2017) 17:281. doi: 10.1186/s12888-017-1440-x, PMID: 28826398 PMC5563911

[42] Tek S Landa RJ. Differences in autism symptoms between minority and non-minority toddlers. J Autism Dev Disord. (2012) 42:1967–73. doi: 10.1007/s10803-012-1445-8, PMID: 22271196 PMC3402594

[43] Pham AV Charles LC. Racial disparities in autism diagnosis, assessment, and intervention among Minoritized youth: sociocultural issues, factors, and context. Curr Psychiatry Rep. (2023) 25:201–11. doi: 10.1007/s11920-023-01417-9, PMID: 37004631

[44] de Leeuw A Happé F Hoekstra RA. A conceptual framework for understanding the cultural and contextual factors on autism across the globe. Autism Res. (2020) 13:1029–50. doi: 10.1002/aur.2276, PMID: 32083402 PMC7614360

[45] Al-Yateem N Slewa-Younan S Halimi A Saeed SA Tliti D Mohammad M. Prevalence of undiagnosed attention deficit hyperactivity disorder (ADHD) symptoms in the young adult population of the United Arab Emirates: a National Cross-Sectional Study. J Epidemiol Glob Health. (2024) 14:45–53. doi: 10.1007/s44197-023-00167-4, PMID: 38079098 PMC11043292

[46] Stiglic N Viner RM. Effects of screentime on the health and well-being of children and adolescents: a systematic review of reviews. BMJ Open. (2019) 9:e023191. doi: 10.1136/bmjopen-2018-023191, PMID: 30606703 PMC6326346

[47] Olusanya BO Nair MKC. Premature mortality in children with developmental disabilities. Lancet Glob Health. (2019) 7:e1601–2. doi: 10.1016/S2214-109X(19)30419-X, PMID: 31653589

[48] Quinn M Halsey J Sherliker P Pan H Chen Z Bennett DA. Global heterogeneity in folic acid fortification policies and implications for prevention of neural tube defects and stroke: a systematic review. EClinicalMedicine. (2024) 67:102366. doi: 10.1016/j.eclinm.2023.102366, PMID: 38169713 PMC10758734

[49] Rajadurai VS Yip WY Lim JSC Khoo PC Tan ES Mahadev A. Evolution and expansion of newborn screening programmes in Singapore. Singapore Med J. (2021) 62:S26–35. doi: 10.11622/smedj.2021073

[50] Lyall K Croen L Daniels J Fallin MD Ladd-Acosta C Lee BK. The changing epidemiology of autism Spectrum disorders. Annu Rev Public Health. (2017) 38:81–102. doi: 10.1146/annurev-publhealth-031816-044318, PMID: 28068486 PMC6566093

[51] Beyens I Valkenburg PM Piotrowski JT. Screen media use and ADHD-related behaviors: four decades of research. Proc Natl Acad Sci USA. (2018) 115:9875–81. doi: 10.1073/pnas.1611611114, PMID: 30275318 PMC6176582

[52] Khatri RB Assefa Y. Drivers of the Australian health system towards health Care for all: a scoping review and qualitative synthesis. Biomed Res Int. (2023) 2023:6648138. doi: 10.1155/2023/6648138, PMID: 37901893 PMC10611547

[53] Daungsupawong H Wiwanitkit V. Comment on "online interest in ADHD predicts ADHD medication prescriptions in Australia from 2004 to 2023: a time-series analysis revealing COVID-19-related acceleration". Australas Psychiatry. (2025) 33:579. doi: 10.1177/10398562251328153, PMID: 40127452

[54] de Vries PJ. Thinking globally to meet local needs: autism spectrum disorders in Africa and other low-resource environments. Curr Opin Neurol. (2016) 29:130–6. doi: 10.1097/WCO.0000000000000297, PMID: 26886354

[55] Lowther C Valkanas E Giordano JL Wang HZ Currall BB O'Keefe K. Systematic evaluation of genome sequencing for the diagnostic assessment of autism spectrum disorder and fetal structural anomalies. Am J Hum Genet. (2023) 110:1454–69. doi: 10.1016/j.ajhg.2023.07.010, PMID: 37595579 PMC10502737

[56] GBD 2021 Fertility and Forecasting Collaborators. Global fertility in 204 countries and territories, 1950-2021, with forecasts to 2100: a comprehensive demographic analysis for the global burden of disease study 2021. Lancet. (2024) 403:2057–99. doi: 10.1016/S0140-6736(24)00550-6, PMID: 38521087 PMC11122687

